# Characterization and Comparison of SLAM/CD150 in Free-Ranging Coyotes, Raccoons, and Skunks in Illinois for Elucidation of Canine Distemper Virus Disease

**DOI:** 10.3390/pathogens9060510

**Published:** 2020-06-24

**Authors:** Caitlin E. Burrell, Chris Anchor, Nadia Ahmed, Jennifer Landolfi, Keith W. Jarosinski, Karen A. Terio

**Affiliations:** 1 Zoological Pathology Program, College of Veterinary Medicine, University of Illinois, Brookfield, IL 60513, USA; nahmed9@illinois.edu (N.A.); landolfi@illinois.edu (J.L.); kterio@illinois.edu (K.A.T.); 2 Forest Preserve District of Cook County, 536 North Harlem Avenue, River Forest, IL 60305, USA; chris.anchor@cookcountyil.gov; 3 Department of Pathobiology, University of Illinois at Urbana-Champaign, Urbana, IL 61802, USA; kj4@illinois.edu

**Keywords:** canine distemper virus, receptor, SLAM/CD150, coyote, raccoon, skunk, wildlife

## Abstract

Canine distemper virus (CDV) is a cause of significant disease in canids and increasingly recognized as a multi-host pathogen, particularly of non-canid families within Carnivora. CDV outbreaks in sympatric mesocarnivores are routinely diagnosed in the Forest Preserve District of Cook County, Illinois. CDV is diagnosed more commonly and the disease more severe in raccoons and striped skunks than in coyotes. Research in other species suggests host cell receptors may play a role in variable disease outcome, particularly, the signaling lymphocyte activation molecule (SLAM) located on lymphoid cells. To evaluate receptor differences, partial SLAM genes were sequenced, and predicted amino acid (AA) sequences and structural models of the proposed viral interface assessed. Of 263 aligned nucleotide base pairs, 36 differed between species with 24/36 differences between canid and non-canids. Raccoon and skunk predicted AA sequences had higher homology than coyote and raccoon/skunk sequences and 8/11 residue differences were between coyote and raccoons/skunks. Though protein structure was similar, few residue differences were associated with charge and electrostatic potential surface alterations between canids and non-canids. RNAScope^®^(Advanced Cell Diagnostics, Silicon Valley, USA) ISH revealed low levels of expression that did not differ significantly between species or tissue type. Results suggest that differences in host receptors may impact species-specific disease manifestation.

## 1. Introduction

Canine distemper virus (CDV), a morbillivirus in the family *Paramyxoviridae*, has long been established as a cause of significant, often fatal, disease in canid species. Other notable morbilliviruses include measles virus, peste-des-petits-ruminants virus (small ruminant morbillivirus), cetacean morbillivirus, phocine distemper virus, and rinderpest virus. CDV has been increasingly recognized as a multi-host pathogen with frequent reports in wildlife populations [1,2,3] and is considered an especially important pathogen in domestic and free-ranging species within the order Carnivora. Potentially of more significance are few individual reports in nonhuman primates, prompting the concern for zoonotic disease potential [4]. The viral and host factors important in species susceptibility and infection outcomes are not currently understood and are an active area of research.

Phylogenetic analysis of the CDV viral hemagglutinin gene, which is generally considered to be the most variable and responsible for encoding a vital protein for initiating infection and viral replication, has been a research focus. Variations in this gene are thought to be important factors impacting species susceptibility. Mutation in the gene at position 549, with substitution of tyrosine for histidine (Y549H), has been implicated in increased infectivity of non-canid species, particularly felids [5,6] while other studies have shown no bias at this site [1,7]. Epidemiologic investigations of wildlife cases have identified numerous strains and distinct CDV lineages in circulation in domestic and free-ranging carnivore populations [8,9,10,11].

At the initiation of infection, the CDV H protein (encoded by the H gene) facilitates binding to the host cell surface receptor and triggers fusion of viral and cellular membranes through the action of viral fusion (F) protein [12,13]. Host infection is initiated at the respiratory mucous membranes via inhalation of virus particles and is followed by replication in regional lymphoid tissue with subsequent spread to the thymus, spleen, bone marrow, and distant lymph nodes through primary viremia. Further viral replication and secondary viremia result in dissemination to the lower respiratory, gastrointestinal, urinary, and central nervous systems [14]. Pathogenesis and clinical signs are the result of viral cell tropism for both host immune and epithelial cells. CDV infection of immune cells is facilitated by viral binding to the signaling lymphocyte activation molecule (SLAM) or CD150 located on thymocytes, activated T and B lymphocytes, dendritic cells, and macrophages, while infection of epithelial cells occurs following viral binding to a different host cell receptor, Nectin-4 [15,16]. In addition, a potential third receptor type located solely on astrocytes has been proposed [17]. Of these receptors, the lymphoid cell receptor SLAM has been implicated in morbillivirus host tropism based on variations of amino acid residues [18,19].

The SLAM receptor is expressed on lymphoid cells in mammalian species, including humans. This receptor is involved in modulation of the innate and acquired immune response through cellular activation of T cells and regulation of natural killer and dendritic cells. The SLAM receptor is a transmembrane protein with two extracellular immunoglobulin superfamily V- and C-type domains. Modeling in marine mammals shows the V domain is composed of 6 beta strands, 5 of which are thought to be the morbillivirus interface [18].

Studies utilizing amino acid sequencing and protein modeling of the proposed viral binding interface of SLAM receptors in morbillivirus-susceptible species have also identified phylogenetic host species variations with the potential to influence viral binding affinity [6,18,19,20,21]. While protein sequences of some non-canid caniforms have been reported, additional evaluation of these receptors is lacking. Due to the potential for differences in CDV infectivity and pathogenicity based on viral-host cell interactions, further investigation into host cell receptors in a wider variety of CDV-susceptible carnivores is warranted.

CDV infection outbreaks in multiple sympatric carnivore species are routinely diagnosed in the Illinois Forest Preserve District of Cook County (FPDCC), the largest forest preserve district in the United States of America. During these outbreaks, disease manifestation has varied based on species; severe disease has been most frequently noted in raccoons (*Procyon lotor*) and striped skunks (*Mephitis mephitis*), while occasionally seen in coyotes (*Canis latrans*). Comparative lack of disease in coyotes is notable given the intense surveillance of radio-collared coyotes in Cook County (Basics of studying coyotes, https://urbancoyoteresearch.com/coyote-info/basics-studying-coyotes) and documented high CDV seroprevalence in this species (C. Chu, personal communication). High CDV seropositivity and an absence of fatal disease has also been noted in coyotes elsewhere suggesting frequent exposure without significant morbidity or mortality [22,23,24]. These findings suggest that pathogenicity is not determined solely by viral intrinsic factors and that host-virus interactions likely play a role in this population. One such potential interaction is that of the virus with host cell receptors required for infectivity and/or viral dissemination. Therefore, the aim of this study was to characterize SLAM receptors in multiple CDV-susceptible sympatric carnivore species within the FPDCC system to identify potential interspecies differences in viral-host interaction.

## 2. Results

### 2.1. SLAM/CD150 DNA and Predicted Amino Acid Sequences

There was 100% similarity among the DNA sequences between tissues within the same individual and between DNA consensus sequences among individuals from the same species. When compared to the coyote sequence, the raccoon sequence had 92.3% homology and the skunk sequence had 91.6% homology. When compared to each other, raccoon and skunk sequences had 96.1% homology. Of the 263 aligned base pairs, 36 differed between species and 24/36 of these differences occurred between coyote and raccoon/skunk sequences. Predicted amino acid sequences covered 86 residues of the binding site from 51–137 [19]. Raccoon and skunk aa sequences had 96.5% homology; raccoon and coyote aa sequences had 89.5% homology, and skunk and coyote aa sequences had 88.3% homology. When compared to the domestic dog (AYM26488.1), homologies were 100%, 87.3%, and 88.5% for coyote, skunk, and raccoon, respectively. Of a total of 86 residues, 11 differed between species, and eight of the differences were between coyote and raccoon/skunks (Table 1). Residues S55, V62, V74, T97, S104, G106, K112, and R129 in the raccoon and skunk were identical to residues in other non-canid Caniformia species [19].

### 2.2. Homology Modeling and Electrostatic Potential Mapping

**Figure 1:**
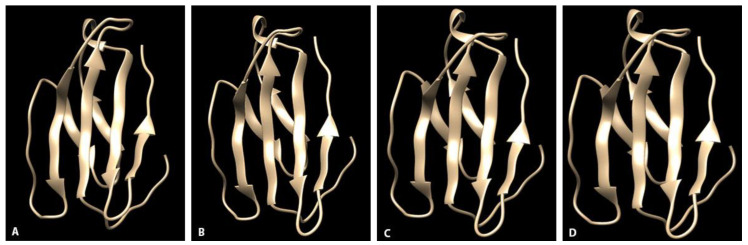
Three-dimensional ribbon models of the signaling lymphocyte activation molecule (SLAM) receptor proposed canine distemper virus (CDV) viral interface. Structures consisted of central anti-parallel β strands, four along the front of the interface and two along the back, and loop structures connecting strands. (**A**) Domestic dog (AYM26448.1). (**B**) Coyote. (**C**) Skunk. (**D**) Raccoon.

**Figure 2:**
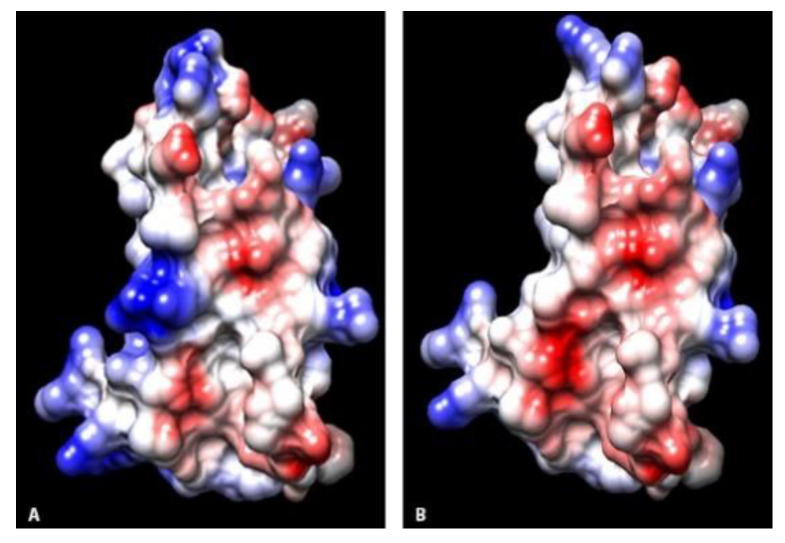
Electrostatic potential maps of the SLAM receptor proposed CDV viral interface along the back β-strands. (**A**) Coyote. (**B**) Raccoon. Blue coloration indicates basic charged regions. Red coloration indicates acidic charged regions.

All amino acid sequences were modeled against the template the measles virus H protein bound to SLAM, B chain (c3alxB) with 100% sequence coverage, 100% confidence, and 62% identity (i.d.). Structures consisted of central anti-parallel β strands, four along one aspect of the interface (front) and two along the opposite aspect (back), and loop structures connecting strands similar to those previously published [18,19]. The basic structure of the SLAM receptor viral interface did not differ among species (Figure 1). In comparison to canids, of the noted substitutions, R55S, K97T, and R106G resulted in charge alterations from a basic or negative charge in canids to a neutral charge in raccoons and skunks. Residue clustering resulted in a change in electrostatic surface potentials between canids and non-canids at these locations. Substitution Q129R resulted in an alteration from a neutral charge in canids to a basic charge in raccoons and skunks. Additionally, substitution H130N in skunks resulted in change from a basic charge in canids and raccoons to a neutral charge in skunks. Residues 97 and 106, which had differences in charge among species, were located on side-chains along the two back β strands and formed a small cluster with other residue side chains along the back of the viral interface in each species. In the domestic dog and coyote electrostatic potential surface maps, this portion of the interface was less positively charged than that in skunks and raccoons (Figure 2). At residue 129, electrostatic surface potentials were overall similar between canids and non-canids.

### 2.3. SLAM/CD150 mRNA Expression

**Figure 3:**
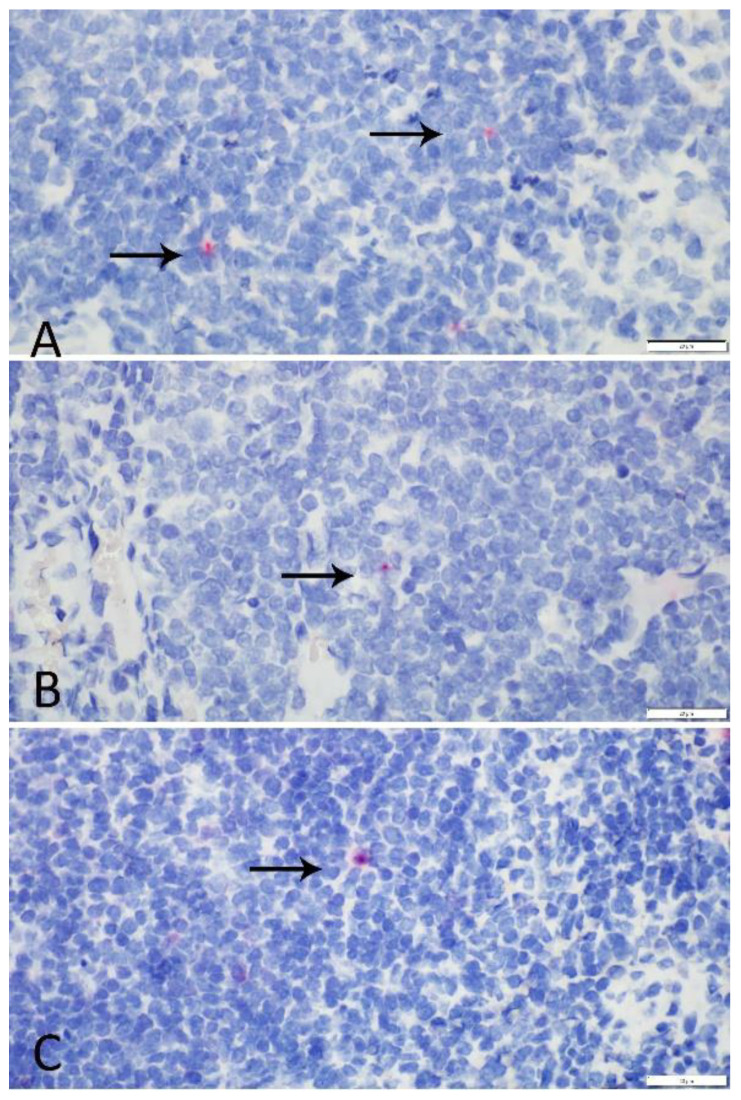
RNAscope^®^ in situ positive hybridization signal. Magenta punctate structures indicate positive signaling and are denoted by arrows. (**A**) Coyote, lymph node, 60×. (**B**) Raccoon, tonsil, 60×. (**C**) Skunk, spleen, 60×.

Expression of SLAM receptor mRNA, as evidenced by positive RNAScope^®^ in situ hybridization (ISH) signal, was detected at some level in at least one representative tissue from each animal (Figure 3). Positive hybridization signal was overall low (<10%/HPF). Mean expression and standard deviation in each tissue per species and species total were calculated (Table 2). No statistically significant variations in mRNA expression (as indicated by ISH signal) were detected between tissue type or species. Expression in the tonsil was highest in the coyote and raccoon. Expression in skunk tonsils could not be evaluated due to lack of adequate samples. In the skunk, expression was highest in the spleen; spleen had the second highest signal in the coyote and the raccoon. Expression was low in the lymph node in all species: coyote, raccoon, and skunk. One raccoon had elevated expression in the spleen and tonsil resulting in a large standard deviation in raccoon tonsil expression. Slight elevations in expression were also noted in tonsillar tissue in one coyote and tonsillar and lymph node tissue in another coyote, as well as splenic expression in one skunk. Tonsil values for the skunk were not included due to the limited sample size. There was no evidence of SLAM RNA expression in epithelial, endothelial, smooth muscle, or fibroblasts in examined tissues.

## 3. Discussion

In this study, SLAM receptor protein structure and mRNA expression distribution were evaluated to better understand potential interspecies differences that may account for variable disease manifestations between sympatric canid and non-canid carnivores (raccoons and skunks) inhabiting the FPDCC Illinois. Through long term, active surveillance, more severe disease has been recognized in raccoons and skunks as compared with coyotes, despite serologic evidence indicating frequent viral exposure and infection in coyotes. To date, much of the published research regarding CDV host susceptibility has focused on virus features or host receptor differences between canids and felids with lesser consideration into canid and non-canid caniforms. Our aim here was to evaluate interspecies differences in sympatric non-canid caniforms.

Amino acid sequences of the CDV-receptor interface were overall similar amongst the study animals with 96.5% homology between non-canids and 88–89.5% homology between coyotes and non-canids. Sequences were similar to published sequences in other carnivore species. Of the few residue alterations noted, differences were most prevalent between coyotes and raccoons/skunks. Higher similarity between these two species when compared to coyotes was not unexpected due to the closer phylogenetic relationship of raccoons and skunks, both of the superfamily *Musteloidea*. One of these changes, residue Q129 in coyotes and R129 in raccoons and skunks, was at a suspected site for viral H protein binding [18], and resulted in alteration of the charge from neutral in coyotes to basic in raccoons and skunks. Basic charged residues in viral-receptor interactions have been shown to have enhanced viral binding affinity [16]. At the adjacent residue proposed to be in this same binding site, skunks had N130 compared to H130 in other study animals, changing from a basic to neutral charge. As H protein binding initiates F protein fusion and viral cell entry, changes in the initial binding affinity may alter that cascade.

Residues at sites 55, 97, and 106 within the viral binding interface had evidence of a change in electrostatic potential between canids and non-canids. These locations have been implicated in stability interface structure [18], and alterations could potentially influence the efficacy of viral binding. In addition, there were several residue alterations that did not result in changes in the charge, including I62 in coyotes and V62 in raccoons and skunks. Residue differences at sites 60, 61, and 63 have been shown to alter host viral susceptibility in human-mouse SLAM chimeras infected with measles virus [25]. Additionally, similarities at site 63 were frequently noted in marine and terrestrial mammals susceptible to the same morbillivirus [18]. While residue substitutions at site 62 were not noted in these previous studies, the proximity of site 62 to residues 60, 61, and 63 suggests site 62 alterations may be a factor influencing differences in disease manifestation in raccoons and skunks as compared to coyotes. Alterations unique to skunks (A79) and raccoons (M79) may also alter viral binding in ways not yet determined.

All animals had evidence of SLAM mRNA expression in mononuclear cells and no apparent expression in epithelial, endothelial, or other mesenchymal cells. Expression did not differ significantly among tissues or between species. Given the absence of significant variation in SLAM mRNA expression between tissue types and species, study results suggested that receptor distribution was not likely the sole factor in species-specific disease manifestation observed in this population. In our RNAscope^®^ ISH study, two coyotes, one raccoon, and one skunk exhibited slightly elevated expression in one or more tissues. These animals had no histologic or gross lesions indicative of CDV infection, and the presence of viral antigen could not be confirmed using immunohistochemical stains in these cases. Though upregulation of SLAM receptors has been demonstrated in cases of CDV infection in domestic dogs [26], increased expression noted in the study tissues was presumably the result of generic antigen stimulation and receptor upregulation. Free-ranging species are exposed to a multitude of potential immune stimulants that could have been contributory. Based on the study presented here, RNAscope^®^ ISH could be a useful technique for future studies investigating the potential for SLAM receptor variation and upregulation in CDV infected wildlife species.

In analyzing the SLAM receptor as a possible contributor to CDV infection pathogenesis, one limitation of the current study was that protein modeling only included the isolated SLAM receptor V region. The absence of the entire sequence could lend itself to protein folding that does not reflect the in vivo receptor structure. However, modelling showed 62% i.d. with the template, representing a high accuracy model, according to program specifications. Another potential limitation was evaluation of mRNA as opposed to protein expression. Though mRNA expression is generally correlative to protein level, detection may not guarantee receptor expression on the cellular membrane. Immunohistochemical staining using antibodies against the SLAMF1 gene validated in humans and rodents was attempted to identify receptor expression; however, adequate immunoreactivity was not observed in the tissues examined (C. Burrell, data not reported). Lastly, due to the opportunistic nature of sampling, study sample size was small. While the SLAM receptor V domain protein structure is unlikely to significantly vary given the described 100% homology within species, a larger study population may be required to confirm findings regarding mRNA expression distribution and exclude receptor expression as a possible contributing factor to disease manifestation. Inclusion of other, less frequently encountered CDV-susceptible species residing in the FPDCC, such as foxes and mink, may also further elucidate CDV epidemiology in this specific population.

To further evaluate the potential significance of the described residue variations and the role of SLAM protein structure in varying disease manifestation, future studies could include in vitro studies employing cell lines with manipulation of amino acid residues to compare viral affinity or using cultured virus to evaluate binding kinetics. Additionally, such studies could also facilitate an assessment of SLAM/CD150 receptor upregulation with CDV infection providing insight into host immune response or mechanisms of viral cell-to-cell spread. Further investigation of intracellular pathways following viral binding is also warranted as the combination of receptors and downstream intracellular mechanics has been implicated as a contributing factor to the host range in measles virus infections [27]. It is interesting to note that coyote amino acid sequences in this study had 100% homology with domestic canine sequences, yet equivalent severe disease in coyotes is not the norm despite serologic evidence of consistent exposure and infection in the FPDCC population. Intracellular mechanisms as previously mentioned, as well as other unidentified host and environmental factors, could potentially be contributing factors to disease development.

The role of Nectin-4, the epithelial cell receptor, in CDV infection of these hosts should also be investigated. Evaluation of Nectin-4 protein structure was attempted for this study, however, amplification of DNA and RNA sequences was unsuccessful using published and de novo designed consensus primer; evaluation of distribution was therefore not pursued. Epithelial cell infection is considered necessary for manifestation of clinical disease in CDV infections and characterization of the receptor in these species may further elucidate disease pathogenesis. While protein structure of Nectin-4 is generally considered to be well-conserved across different species [28], differences in cellular expression and distribution could potentially contribute to disease manifestation.

In conclusion, there are multiple factors that could influence the variable clinical expression of canine distemper virus infection, and disease manifestation is likely the result of a combination of viral, host, and environmental factors. The data presented here suggest that differences in host receptors may potentially account for some of the variation in disease expression in the various mesocarnivores in the FPDCC.

## 4. Materials and Methods

### 4.1. Animals and Tissue Sampling

Fresh carcasses were submitted from the FPDCC to the University of Illinois Zoological Pathology Program for diagnostic evaluation or routine disease surveillance from December 2018 through April 2019. Animals were captured and immediately humanely euthanized or found dead with minimal post-mortem autolysis. A full necropsy with histologic examination was performed. Individuals with a history of neurologic signs, gross or microscopic lesions suggestive of CDV or evidence of viral antigen immunoreactivity using immunohistochemical stains were excluded. Study animals included an equal number of coyotes (n = 5), raccoons (n = 5), and striped skunks (n = 5). Both sexes were represented, and all animals were juveniles and adults although exact ages of individuals were unknown. Standardized samples of cerebrum, lung, spleen, retropharyngeal and/or cervical lymph node, stomach, and urinary bladder were collected and stored at −80 °C until prepared and tested for molecular analysis. Additional separate standardized samples of retropharyngeal and/or cervical lymph node, spleen, and tonsil were preserved in 10% neutral buffered formalin for 24–72 h prior to transfer to 70% ethyl alcohol and routine processing and paraffin embedding.

### 4.2. DNA Extraction and SLAM/CD150 Amplification and Sequencing

DNA extraction using 25 mg each of lymph node, lung, stomach, brain, and urinary bladder and 10 mg of spleen was performed for each case per manufacturer’s instructions using Qiagen DNeasy Blood and Tissue Kit (Qiagen, GmBH, Qiagen Strasse 1, 40724, Hilden, Germany). PCR was performed on extracted DNA using previously published primers to amplify sequences from the SLAM receptor V domain which contains the proposed interface of the SLAM receptor and CDV [19]. The predicted 354 bp fragment was amplified in coyotes using primers SLAM-DF2 and SLAM-DR2. The same primers failed to amplify DNA from raccoon and skunk tissues. For these species, a 263 bp fragment was amplified using primers Car-F1 and SLAM-DR2 [19]. Conventional PCR reactions included 10× reaction buffer with MgCl_2_, AmpliTaq Gold polymerase (Applied Biosystems by Thermo Fisher Scientific, Waltham, MA, USA), dNTP mix (Thermo Scientific, Waltham, MA, USA), 6.25 pmol of each primer and distilled water to final reaction volume of 25 µL. Thermocycle reaction parameters were the following: initial denaturation at 95 °C for 10 min, followed by 35 cycles of 94 °C for 1 min, 55 °C for 1 min, and 72 °C for 1 min, concluded by final extension at 72 °C for 5 min. Distilled water was amplified as a negative control; after initial fragment sequencing, aliquots of coyote spleen extracted DNA were used as a positive control. Amplification of DNA was confirmed by agarose gel electrophoresis. Amplicons were purified using ExoSAP-IT TM PCR Product Cleanup Reagent (Applied Biosystems by Thermo Fisher Scientific, Waltham, MA, USA) and directly sequenced using an automated sequencer at a subcontracted laboratory (University of Chicago Comprehensive Cancer Center DNA Sequencing and Genotyping Facility). Similarity to published domestic canid, CD150/SLAM sequences was determined on select sequences from all three study species using NCBI Basic Local Alignment Search Tool (BLAST). DNA sequences were aligned using Geneious prime 2019.1.3 software to create consensus sequences at the individual then species level. Coyote, raccoon, and skunk consensus sequences were aligned to identify species-specific nucleotide discrepancies. Predicted amino acid sequences were produced from DNA sequences and aligned with a published domestic canine SLAM receptor amino acid sequence (GenbankAYM26448.1) and distance matrices calculated. Polarity and charge alterations were determined for each differing residue manually.

### 4.3. Homology Modeling

Predicted amino acid sequences for each individual and the published domestic canine SLAM (Genbank AYM26448.1) and isolated V region sequences were uploaded to a PHYRE2 web-based modeling program [29]. Program database (PDB) files were uploaded to the Chimera molecular modeling system [30]. Three-dimensional ribbon diagrams of the protein structures at the interface surfaces were visually compared across species. Using the Chimera modeling system, amino acid residues were labelled and electrostatic potential surface maps calculated for each protein model.

### 4.4. RNA Expression Demonstrated by RNAscope^®^ In Situ Hybridization

Formalin-fixed tissues were processed routinely. Samples of spleen, tonsil, and retropharyngeal or cervical lymph node were sectioned at 5 µm and mounted on StarFrost plus coated microscope slides (ProSciTech Pty. Ltd., Thuringowa Central, QLD, Australia 4817). RNAscope^®^ ISH chromogenic manual assay was performed using the RNAscope^®^ 2.5 HD Detection Kit Red (Advanced Cell Diagnostics, Inc., Newark, CA, USA) and a 19-pair oligonucleotide probe targeted against the signaling lymphocytic activation molecule family member 1 (SLAMF1) in the dog, ferret, and seal (16s rRNA) (ACD # 452151) according to the manufacturer’s directions. This probe has homology to the coyote, raccoon, and skunk sequences described previously. Formalin-fixed, paraffin-embedded (FFPE) unstained sections of domestic ferret lymph node was used as a positive control. A negative control probe targeted against the DapB gene from the *Bacillus subtilis* strain SMY was used on all sections in parallel with the target probe. Hybridization signal in mononuclear cells in equivalent tissues was quantitatively evaluated using light microscopy. Spleen, lymph node, and tonsil was evaluated from each individual: in coyotes, spleen (n = 5), tonsil (n = 5), and lymph node (n = 5); in raccoons, spleen (n = 5), tonsil (n = 5), and lymph node (n = 5); and in skunks, spleen (n = 5), tonsil (n = 1), and lymph node (n = 5). Tonsil was not available for evaluation in 4/5 skunks. The total number of cells with positive hybridization signal from 10 high-powered (400×) adjacent fields was calculated for each tissue by a single observer (C. Burrell). Evaluation was confined to the cortices in tonsil and lymph node and periarteriolar lymphoid sheaths in spleen. An ANOVA test was performed to compare tissue-specific and total RNA expression across species. An independent t-test was performed to assess canid vs non-canid tissue-specific and total RNA expression. *p* values less than 0.05 were considered statistically significant.

## Figures and Tables

**Table 1:** Comparison of the amino acid residues of the CDV interface on the SLAM receptor in mesocarnivores of the FPDCC. Interspecies residue differences are highlighted in grey. Underlined sites (55, 97, 106, 129, 130) represent residue alterations that result in charge differences.

	Amino Acid Residue Comparison																						
**Species**	**55**	**60**	**61**	**62**	**63**	**65**	**70**	**71**	**74**	**75**	**79**	**85**	**90**	**95**	**97**	**100**	**104**	**106**	**110**	**112**	**120**	129	130
Domestic dog (AYM26448.1)	R	I	H	I	L	T	P	G	I	K	V	E	R	G	K	L	T	R	S	R	M	Q	H
Coyote	R	I	H	I	L	T	P	G	I	K	V	E	R	G	K	L	T	R	S	R	M	Q	H
Raccoon	S	I	H	V	L	T	P	G	V	K	M	E	R	G	T	L	S	G	S	K	M	R	H
Skunk	S	I	H	V	L	T	P	A	V	K	V	E	R	G	T	L	S	G	S	K	M	R	N

**Table 2:** Comparison of the SLAM positive hybridization signal in the spleen, tonsil, and lymph node of mesocarnivores of the Forest Preserve District of Cook County (FPDCC). Numbers indicate positive signal/10 high-powered fields (40×).

	Coyote					Raccoon				Skunk			
	Spleen	Tonsil	Lymph Node			Spleen	Tonsil	Lymph Node			Spleen	Lymph Node	
**1**	7	7	4		**1**	3	2	1		**1**	5	6	
**2**	10	23	1		**2**	5	6	11		**2**	8	4	
**3**	11	14	25		**3**	7	7	2		**3**	1	8	
**4**	3	2	0		**4**	5	1	5		**4**	20	4	
**5**	1	4	0		**5**	15	111	2		**5**	0	4	
**Mean**	6.40	10.00	6.00			7.00	25.40	4.20			6.80	5.20	
**Std dev**	4.34	8.57	10.75			4.69	47.92	4.09			8.04	1.79	
**Species total**				7.47					12.2				6

## References

[1] Terio K.A., Craft M.E. (2013). Canine distemper virus (CDV) in another big cat: Should CDV be renamed carnivore distemper virus?. mBio.

[2] Beineke A., Baumgärtner W., Wohlsein P. (2015). Cross-species transmission of canine distemper virus—An update. One Health.

[3] Feng N., Yu Y., Wang T., Wilker P., Wang J., Li Y., Sun Z., Gao Y., Xia X. (2016). Fatal canine distemper virus infection of giant pandas in China. Sci. Rep..

[4] Zhao J.-J., Yan X.-J., Chai X.-L., Martella V., Luo G.-L., Zhang H.-L., Gao H., Liu Y.-X., Bai X., Zhang L. (2010). Phylogenetic analysis of the haemagglutinin gene of canine distemper virus strains detected from breeding foxes, raccoon dogs and minks in China. Vet. Microbiol..

[5] Nikolin V.M., Wibbelt G., Michler F.-U.F., Wolf P., East M.L. (2012). Susceptibility of carnivore hosts to strains of canine distemper virus from distinct genetic lineages. Vet. Microbiol..

[6] Nikolin V.M., Osterrieder K., von Messling V., Hofer H., Anderson D., Dubovi E., Brunner E., East M.L. (2012). Antagonistic pleiotropy and fitness trade-offs reveal specialist and generalist traits in strains of canine distemper virus. PLoS ONE.

[7] Seimon T.A., Miquelle D.G., Chang T.Y., Newton A.L., Korotkova I., Ivanchuk G., Lyubchenko E., Tupikov A., Slabe E., McAloose D. (2013). Canine distemper virus: An emerging disease in wild endangered Amur tigers (Panthera tigris altaica). mBio.

[8] Martinez-Gutierrez M., Ruiz-Saenz J. (2016). Diversity of susceptible hosts in canine distemper virus infection: A systematic review and data synthesis. BMC Vet. Res..

[9] Techangamsuwan S., Banlunara W., Radtanakatikanon A., Sommanustweechai A., Siriaroonrat B., Lombardini E.D., Rungsipipat A. (2015). Pathologic and molecular virologic characterization of a canine distemper outbreak in farmed civets. Vet. Pathol..

[10] Pope J.P., Miller D.L., Riley M.C., Anis E., Wilkes R.P. (2016). Characterization of a novel canine distemper virus causing disease in wildlife. J. Vet. Diagn. Investig..

[11] Needle D.B., Burnell V.C., Forzán M.J., Dubovi E.J., Schuler K.L., Bernier C., Hollingshead N.A., Ellis J.C., Stevens B.A., Tate P. (2019). Infection of eight mesocarnivores in New Hampshire and Vermont with a distinct clade of canine distemper virus in 2016–2017. J. Vet. Diagn. Investig..

[12] Parida S., Muniraju M., Mahapatra M., Muthuchelvan D., Buczkowski H., Banyard A.C. (2015). Peste des petits ruminants. Vet. Microbiol..

[13] Rendon-Marin S., da Fontoura Budaszewski R., Canal C.W., Ruiz-Saenz J. (2019). Tropism and molecular pathogenesis of canine distemper virus. Virol. J..

[14] Maclachlan N.J., Dubovi E.J. (2010). Fenner’s Veterinary Virology.

[15] Sato H., Yoneda M., Honda T., Kai C. (2012). Morbillivirus receptors and tropism: Multiple pathways for infection. Front. Microbiol..

[16] Loots A.K., Mitchell E., Dalton D.L., Kotzé A., Venter E.H. (2017). Advances in canine distemper virus pathogenesis research: A wildlife perspective. J. Gen. Virol..

[17] Alves L., Khosravi M., Avila M., Ader-Ebert N., Bringolf F., Zurbriggen A., Vandevelde M., Plattet P. (2015). SLAM- and Nectin-4-Independent noncytolytic spread of canine distemper virus in astrocytes. J. Virol..

[18] Ohishi K., Ando A., Suzuki R., Takishita K., Kawato M., Katsumata E., Ohtsu D., Okutsu K., Tokutake K., Miyahara H. (2010). Host–virus specificity of morbilliviruses predicted by structural modeling of the marine mammal SLAM, a receptor. Comp. Immunol. Microbiol. Infect. Dis..

[19] Ohishi K., Suzuki R., Maeda T., Tsuda M., Abe E., Yoshida T., Endo Y., Okamura M., Nagamine T., Yamamoto H. (2014). Recent host range expansion of canine distemper virus and variation in its receptor, the signaling lymphocyte activation molecule, in carnivores. J. Wildl. Dis..

[20] Yadav A.K., Rajak K.K., Bhatt M., Kumar A., Chakravarti S., Sankar M., Muthuchelvan D., Kumar R., Khulape S., Singh R.P. (2019). Comparative sequence analysis of morbillivirus receptors and its implication in host range expansion. Can. J. Microbiol..

[21] Hashiguchi T., Ose T., Kubota M., Maita N., Kamishikiryo J., Maenaka K., Yanagi Y. (2011). Structure of the measles virus hemagglutinin bound to its cellular receptor SLAM. Nat. Struct. Mol. Biol..

[22] Cypher B.L., Scrivner J.H., Hammer K.L., O’Farrell T.P. (1998). Viral antibodies in coyotes from California. J. Wildl. Dis..

[23] Malmlov A., Breck S., Fry T., Duncan C. (2014). Serologic survey for cross-species pathogens in urban coyotes (*Canis latrans*), Colorado, USA. J. Wildl. Dis..

[24] Gese E.M., Schultz R.D., Rongstad O.J., Andersen D.E. (1991). Prevalence of antibodies against aanine parvovirus and canine distemper virus in wild coyotes in southeastern Colorado. J. Wildl. Dis..

[25] Ohno S., Seki F., Ono N., Yanagi Y. (2003). Histidine at position 61 and its adjacent amino acid residues are critical for the ability of SLAM (CD150) to act as a cellular receptor for measles virus. J. Gen. Virol..

[26] Wenzlow N., Plattet P., Wittek R., Zurbriggen A., Grone A. (2007). Immunohistochemical demonstration of the putative canine distemper virus receptor CD150 in dogs with and without distemper. Vet. Pathol..

[27] Iwasaki M., Yanagi Y. (2011). Expression of the Sendai (murine parainfluenza) virus C protein alleviates restriction of measles virus growth in mouse cells. Proc. Natl. Acad. Sci. USA.

[28] Noyce R.S., Bondre D.G., Ha M.N., Lin L.-T., Sisson G., Tsao M.-S., Richardson C.D. (2011). Tumor cell marker PVRL4 (Nectin 4) is an epithelial cell receptor for measles virus. PLoS Pathog..

[29] Kelley L.A., Mezulis S., Yates C.M., Wass M.N., Sternberg M.J.E. (2015). The Phyre2 web portal for protein modeling, prediction and analysis. Nat. Protoc..

[30] Pettersen E.F., Goddard T.D., Huang C.C., Couch G.S., Greenblatt D.M., Meng E.C., Ferrin T.E. (2004). UCSF Chimera--a visualization system for exploratory research and analysis. J. Comput. Chem..

